# Surfactant-Dependent Bulk Scale Mechanochemical Synthesis of CsPbBr_3_ Nanocrystals for Plastic Scintillator-Based X-ray Imaging

**DOI:** 10.1021/acsanm.3c02531

**Published:** 2023-08-07

**Authors:** Joydip Ghosh, Joseph O’Neill, Mateus G. Masteghin, Isabel Braddock, Carol Crean, Robert Dorey, Hayden Salway, Miguel Anaya, Justin Reiss, Douglas Wolfe, Paul Sellin

**Affiliations:** † Department of Physics, University of Surrey, Guildford GU2 7XH, U.K.; ‡ Advanced Technology Institute, University of Surrey, Guildford GU2 7XH, U.K.; § Department of Chemistry, University of Surrey, Guildford GU2 7XH, U.K.; ∥ School of Mechanical Engineering Sciences, University of Surrey, Guildford GU2 7XH, U.K.; ⊥ Department of Chemical Engineering and Biotechnology, University of Cambridge, Cambridge CB3 0AS, U.K.; # Applied Research Laboratory, Materials Science and Engineering Department, The Pennsylvania State University, University Park, Pennsylvania 16802, United States; ¶ Departamento Física de la Materia Condensada, Instituto de Ciencia de Materiales de Sevilla, Universidad de Sevilla−CSIC, Avenida Reina Mercedes SN, Sevilla 41012, Spain

**Keywords:** mechanochemical synthesis, CsPbBr_3_ nanocrystals, strong photoluminescence, plastic scintillator, X-ray imaging

## Abstract

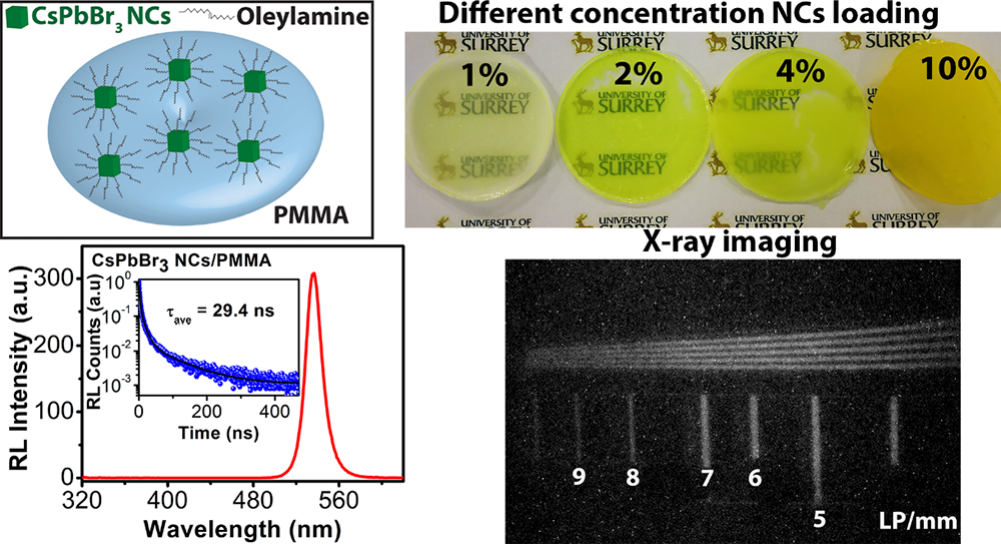

We report a facile, solvent-free surfactant-dependent mechanochemical synthesis of highly luminescent CsPbBr_3_ nanocrystals (NCs) and study their scintillation properties. A small amount of surfactant oleylamine (OAM) plays an important role in the two-step ball milling method to control the size and emission properties of the NCs. The solid-state synthesized perovskite NCs exhibit a high photoluminescence quantum yield (PLQY) of up to 88% with excellent stability. CsPbBr_3_ NCs capped with different amounts of surfactant were dispersed in toluene and mixed with polymethyl methacrylate (PMMA) polymer and cast into scintillator discs. With increasing concentration of OAM during synthesis, the PL yield of CsPbBr_3_/PMMA nanocomposite was increased, which is attributed to reduced NC aggregation and PL quenching. We also varied the perovskite loading concentration in the nanocomposite and studied the resulting emission properties. The most intense PL emission was observed from the 2% perovskite-loaded disc, while the 10% loaded disc exhibited the highest radioluminescence (RL) emission from 50 kV X-rays. The strong RL yield may be attributed to the deep penetration of X-rays into the composite, combined with the large interaction cross-section of the X-rays with the high-Z atoms within the NCs. The nanocomposite disc shows an intense RL emission peak centered at 536 nm and a fast RL decay time of 29.4 ns. Further, we have demonstrated the X-ray imaging performance of a 10% CsPbBr_3_ NC-loaded nanocomposite disc.

## 1 Introduction

Sensitive X-ray detectors are always in high demand in a wide field of applications, including medical imaging, industry, product quality inspection, security checks, scientific research, etc.^1−4^ Indirect scintillator detectors convert high-energy ionizing radiation into ultraviolet (UV) or visible light that can be further detected by a photodetector or imaging sensor.^5^ The X-ray attenuation efficiency, optical quantum yield, and time profile are the most important fundamental parameters that determine scintillator performance. Thallium-doped cesium iodide (CsI:Tl), terbium-doped gadolinium oxysulfide (Gd_2_O_2_S:Tb, GOS), cerium-activated YAlO_3_ (YAlO_3_:Ce), and cerium-doped Lu_2_SiO_5_ (LSO) are widely used as commercial scintillators.^6,7^ However, these commercial scintillators have certain limitations and shortcomings due to their high-temperature growth, complicated manufacturing technologies, high cost, high power consumption, etc. Therefore, it is advantageous to develop alternative low-temperature and solution-processable scintillating materials for sensitive X-ray detection.

Recently, metal halide perovskites have attracted a lot of attention due to their low-cost solution processable fabrication and impressive optoelectronic properties, with high X-ray absorption coefficient, large electron–hole diffusion lengths, tunable emission wavelength, etc.^8−14^ All-inorganic metal halide perovskites including CsPbBr_3_ nanocrystals (NCs) exhibit an excellent range of optical properties including high photoluminescence quantum yield (PLQY), narrow PL full-width at half-maxima (FWHM), high photostability, and superior performance as light-emitting diodes (LEDs).^15^ Recently, all-inorganic perovskite NCs have emerged as promising scintillating materials due to their efficient radioluminescence (RL) emission, high X-ray attenuation, and low detection limit.^16−23^ The nanocomposite plastic scintillator, in which high-Z nanoparticles with high emission are added to a plastic, has great potential for ionizing radiation detection and imaging.^24^ However, these NCs are difficult to cast uniformly into compact solid or composite films, and emission quenching is often observed due to self-assembly and spontaneous aggregation, particularly at high mass loading.^25^

A variety of synthesis methods, such as hot injection, anion exchange, solvothermal, re-precipitation, microwave-assisted synthesis, etc., have been reported for the growth of highly luminescent CsPbBr_3_ NCs.^26−28^ However, these synthesis strategies involve high reaction temperatures, the use of toxic volatile organic solvents, and complicated experimental procedures and purification techniques. Previously, we reported the synthesis of color-tunable all inorganic perovskite NCs using a nearly solvent-free surfactant-assisted ball milling method for applications in color-tunable LEDs.^29,30^ However, a systematic surfactant-dependent study of morphology and optical properties of the NCs by the mechanochemical synthesis method is required. The use of perovskite NCs produced by a facile solid-state method for ionizing radiation detection is yet to be explored.

Here, we report a facile, bulk-scale surfactant-dependent mechanochemical synthesis of highly luminescent CsPbBr_3_ NCs and their application as X-ray scintillators. Our room temperature synthesis method is free of toxic organic and volatile solvents, which is significant when compared to other reported synthesis techniques. The synthesis method requires minimal postprocessing (i.e., without centrifugation, washing, and redispersion processes) while producing remarkably efficient X-ray scintillation. A small amount of the surfactant oleylamine (OAM) play an important role in the two-step ball milling method to control the size and emission properties of the NCs.^31^ The solid-state synthesized perovskite NCs exhibit high PLQY of up to 88%. CsPbBr_3_ perovskite NC dispersions capped with different amounts of surfactant were mixed with PMMA plastic and cast into discs of 2 mm thickness. By increasing the OAM concentration during synthesis, the PL yield of the CsPbBr_3_/PMMA nanocomposite was increased, which is attributed to the reduced aggregation and PL quenching of the NCs.^25^ We also varied the perovskite loading concentration in the nanocomposite and studied the resulting PL and RL emission properties. We observed the highest PL emission with the 2% perovskite-loaded PMMA disc, while the 10% CsPbBr_3_/PMMA nanocomposite disc exhibited the highest RL emission from 50 kV X-rays. The strong RL light yield may be attributed to the deep penetration of X-rays into the composite, combined with the large X-ray interaction cross-section of the high-Z atoms within the NCs. The CsPbBr_3_/PMMA nanocomposite disc shows a highly intense RL emission peak at 536 nm with FWHM ∼16 nm and a fast RL decay time of 29.4 ns. Furthermore, we have demonstrated the X-ray imaging performance of a 10% CsPbBr_3_ NC-loaded PMMA nanocomposite disc, which exhibits high spatial resolution.

## 2 Experimental Details

### 2.1 Materials

Lead (II) bromide (PbBr_2_, >98%, Sigma Aldrich), cesium bromide (CsBr 99.999%, Sigma Aldrich), OAM (>98%, Sigma Aldrich), and poly(methyl methacrylate) (PMMA) powder (Sigma Aldrich) were used as precursor materials. All the chemicals were used as-received without any further purification.

### 2.2 Mechanochemical Synthesis of CsPbBr_3_ NCs

**Scheme 1:**
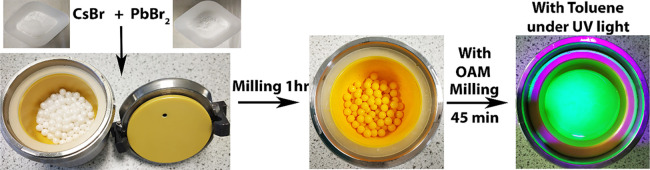
Photographs of the Solid-State Synthesis Steps of CsPbBr_3_ NCs by Ball Milling Method

0.5 mM of CsBr and 0.5 mM of PbBr_2_ were loaded with 35 g of 5 mm diameter zirconium oxide balls in a 45 mL zirconium oxide jar under ambient conditions and milled for 1 h at a rotation speed of 400 rpm in a planetary ball mill (Planetary Micro Mill PULVERISETTE 7) which produces a fine powder of CsPbBr_3_. Next, different amounts of OAM (0, 0.05, 0.1, 0.2, 0.4, and 0.8 mL) were added to the milling vial and milled for another 45 min to study the effect of the surfactant in the formation of CsPbBr_3_ NCs. Following this, the synthesized product was dispersed in 15 mL of toluene. The solution was allowed to settle for 24 h, at which point the precipitate was separated and the colloidal dispersion of all-inorganic CsPbBr_3_ perovskite NCs was stored for further use. The synthesis steps of the perovskite NCs are shown in Scheme 1.

### 2.3 Nanocomposite Fabrication

1.5 g of PMMA powder was mixed with the desired amount of CsPbBr_3_ NCs dispersed in toluene and mixed by vigorous stirring and heating at 60 °C for 24 h. Approximately 60% of the toluene was allowed to evaporate by heating to increase the concentration of the dispersion. Then, the dispersion was poured into PTFE molds and left to harden overnight.

### 2.4 Characterization Techniques

The morphology and crystal structure of CsPbBr_3_ NCs synthesized with different amounts of OAM were analyzed using a Talos F200i 200 kV transmission electron microscope (TEM) (Thermo Fisher Scientific) and a Titan3 G2 60–300 kV TEM (ThermoFisher Scientific). Photoluminescence (PL) spectra were recorded with 405 nm laser excitation using a QE 6500 spectrometer (Ocean Insight), and a 420 nm long pass filter. The low-temperature PL measurement of CsPbBr_3_/PMMA nanocomposites was performed using a cryostat stage connected to a liquid nitrogen supply and a heating plate with the temperature set using a temperature measurement control unit TIC 304-MA (CryoVac). The UV–Vis transmittance and absorption spectra of the samples were acquired using a UV-2401PC spectrophotometer (Shimadzu). Time-resolved PL (TRPL) decay time measurements were performed using a PicoQuant fluorescence lifetime spectrometer (PicoQuant), with the excitation of a 405 nm pulsed laser. The morphology and elemental distribution of CsPbBr_3_/PMMA nanocomposite were studied using a JEOL JSM-7100F scanning electron microscope (SEM). The pulsed RL decay measurements were acquired with a laser-excited 40 kV pulsed X-ray source N5084 (Hamamatsu Photonics) coupled to an FLS1000 Photoluminescence Spectrometer (XS1, Edinburgh Instruments). X-ray sensitivity data were obtained by mounting the samples in a dark box using a Mini-X2 X-ray tube (Amptek) with a tube voltage of 40 kV, adjusting the current between 10 and 200 μA and with light collection via a PMT (ET Enterprises). X-ray images using the nanocomposite scintillator discs were obtained using a Hamamatsu L6732-01 X-ray tube (Hamamatsu Photonics) at 80 kV with a tube current of 100 μA and a commercial optical camera.

## 3 Results and Discussion

### 3.1 Morphology and Structural Analysis

**Figure 1:**
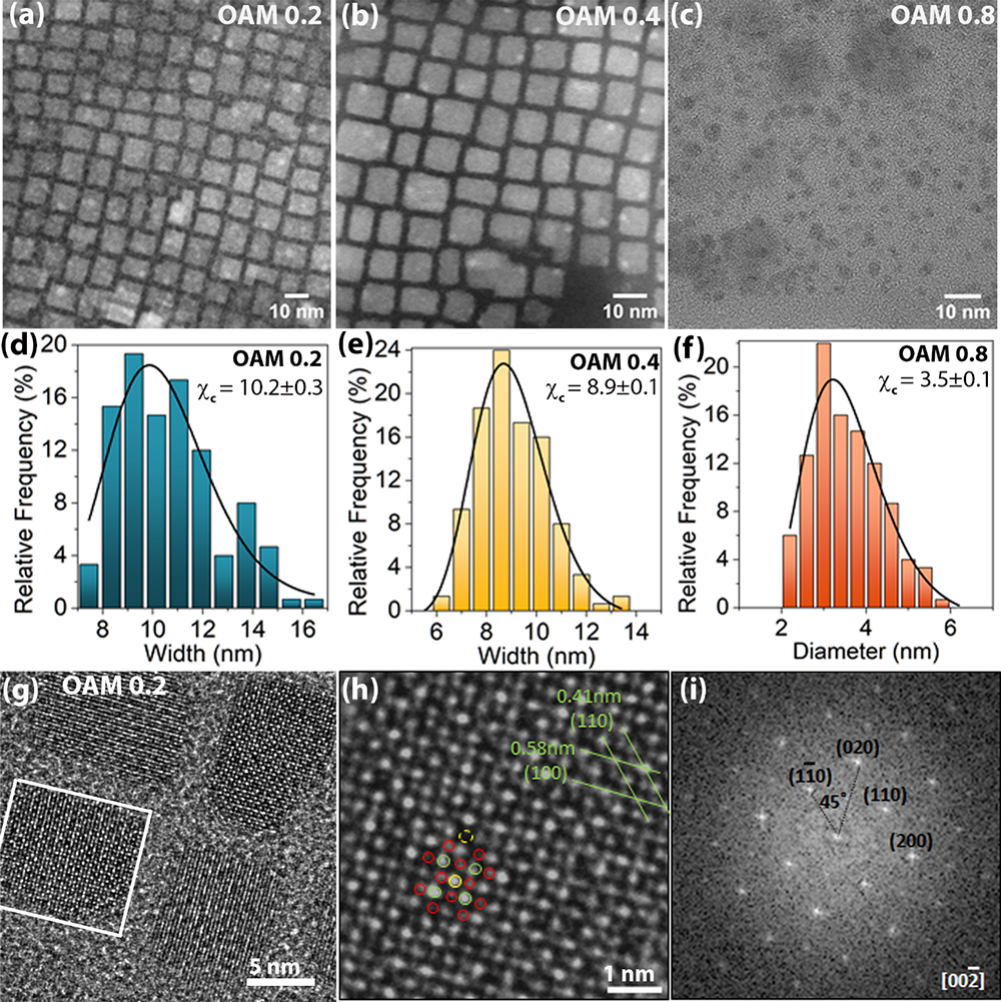
TEM images of CsPbBr_3_ NCs synthesized with (a) 0.2 (b) 0.4, and (c) 0.8 mL of oleylamine. The particle size distribution calculated from TEM images of the sample (d) OAM 0.2 (e) OAM 0.4, and (f) OAM 0.8. (g) High-resolution TEM image OAM 0.2 NCs showing atomic columns. (h) Magnified HRTEM image with atomic resolution. (i) FFT pattern showing the diffraction spots of CsPbBr_3_.

The morphology and structure of CsPbBr_3_ perovskite NCs mechano-synthesized by using different concentrations of OAM were studied using TEM imaging. Figure 1a–c shows TEM images of CsPbBr_3_ NCs synthesized with 0.2, 0.4, and 0.8 mL of OAM, respectively. The TEM images of OAM 0, OAM 0.05, and OAM 0.1 samples are shown in Figure S1(a–c) (Supporting Information), respectively. The CsPbBr_3_ NCs exhibit a cubic-shaped morphology, while in the case of OAM 0.8, smaller-sized spherical CsPbBr_3_ quantum dots were observed. The nanostructures’ dimensions of width (cubes) and diameter (dots) were measured, and the relative frequency distribution was plotted from which a Gaussian fit was used to obtain average particle sizes, as shown in Figures 1d–f and S1d–f (Supporting Information). The average particle sizes were observed to be 36.3, 11.5, 10.6, 10.2, 8.9, and 3.5 nm for samples OAM 0, OAM 0.05, OAM 0.1, OAM 0.2, OAM 0.4, and OAM 0.8, respectively. Some larger size NCs were also observed in samples OAM 0 and OAM 0.05, while NCs were nearly monodispersed in the case of the sample synthesized with a higher amount of OAM. Interestingly, the particle size of the NCs decreased with the increase in OAM concentration. The long-chain amine surfactant OAM plays an important role in the size and optical emission properties of the NCs/QDs.^32,33^ The small amount of OAM surfactant functionalized the surface of the NCs, which led to the strong emission properties. The OAM surfactant dynamically stabilizes the surface of the CsPbBr_3_ NCs via [Br···H–N^+^] hydrogen bonding, i.e., the interactions between the ammonium groups and the edge halide atoms which controls the shape and size.^32,34^ The surfactant capped on the NCs prevents the self-assembly and agglomeration of the NCs in colloidal form which results in superior PL emission and stability.^35^ With the increase in OAM concentration in the liquid-assisted milling, the grinding process may be smoother, which facilitates a decrease in NCs size.^36^ However, the small amount of OAM ligand may play a complicated role in the two-step synthesis process. With the decrease in the NCs size, the PL emission peak and absorption edge were blue-shifted due to the quantum confinement effect (discussed later).

**Figure 2:**
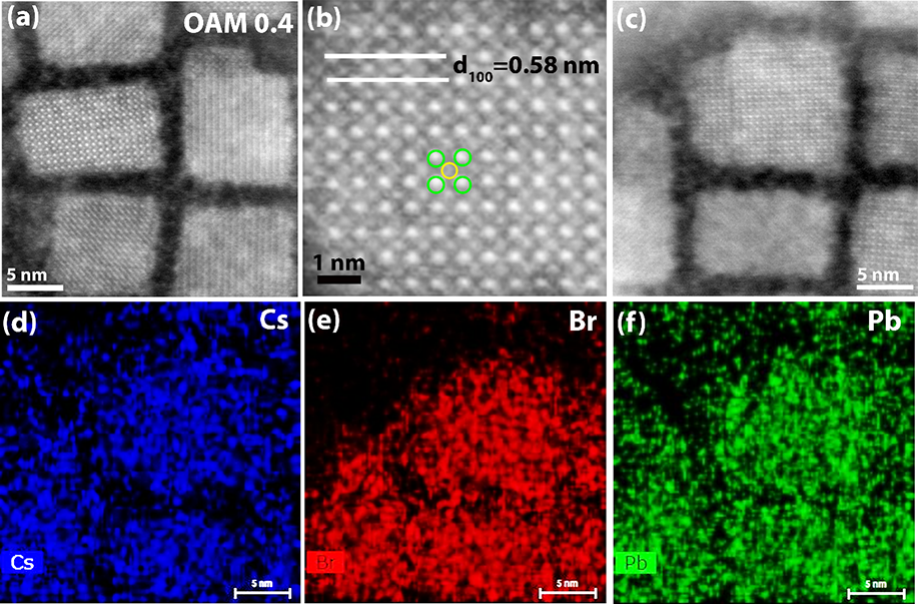
(a) HAADF-STEM image showing atomic columns of OAM 0.4. (b) Magnified HRTEM image with atomic resolution. (c–f) STEM image and corresponding EDS elemental color mapping of Cs, Br, and Pb, respectively.

For a deeper insight into the structure and crystalline quality of CsPbBr_3_ NCs, we performed a high-resolution TEM (HRTEM) analysis of the OAM 0.2 sample, as shown in Figure 1g–h. The NCs exhibit excellent crystalline quality confirming that this nearly solvent-free robust solid-state synthesis method can be a good alternative for the synthesis of highly crystalline perovskite NCs. Figure 1h shows the magnified HRTEM image of Figure 1g, which clearly depicts the crystalline arrangement of atoms in the CsPbBr_3_ crystal. The yellow, green, and red circles in Figure 1h correspond to Cs, Pb, and Br atoms, respectively, confirming the cubic structure of CsPbBr_3_ perovskite. Some Cs vacancies were observed (dotted yellow line), which may be due to the robust mechanosynthesis process, although these appear to have minimal effect on the PL emission which is anticipated because the A site vacancy in halide perovskites has a minimal effect on the energy band structure. The lattice spacing of 0.58 and 0.41 nm for CsPbBr_3_ NCs correspond to (100) and (110) planes, respectively (Figure 1h). The corresponding fast Fourier transform (FFT) of CsPbBr_3_ NCs is presented in Figure 1i, confirming high crystalline quality with a cubic phase of *Pm*-3*m* space group. The diffraction spots are indexed with different crystal planes viewed from the [002̅] zone axis. Figure S2 (Supporting Information) shows the selected area electron diffraction (SAED) pattern of CsPbBr_3_ NCs. The bright rings correspond to (100), (110), (002), (210), and (202) planes. All planes were indexed based on PDF #18-364. Figure 2a presents the high-angle annular dark-field scanning TEM (HAADF STEM) image of OMA 0.4 NCs, while the magnified HRTEM image of NCs is shown in Figure 2b. The yellow and green circles represent Cs and Pb atoms, respectively. Interestingly, the OAM 0.4 sample is more defect-free as compared to OAM 0.2 which may be attributed to the smother milling with a slightly higher surfactant concentration. The nanoscale elemental composition of the perovskite NCs was further confirmed by STEM EDX mapping. Figure 2c shows the TEM image of the NCs, while Figure 2d–f depicts the corresponding elemental mapping Cs, Br, and Pb at high resolution. The EDS images show a uniform stoichiometric distribution across the nanoparticles, with no evidence of significant compositional nonuniformities.

The formation of CsPbBr_3_ without any impurity phase was further confirmed by XRD measurement. The XRD peaks at 2θ values of 15.11°, 21.41°, 30.65°, 34.28°, 37.61°, and 43.70° correspond to (100), (110), (200), (210), (211), and (220), respectively, which match well with the literature (Figure S3, Supporting Information).^37,38^ No peaks associated with CsBr or PbBr_2_ phases were observed confirming full conversion into the perovskite phase without any substantial impurity phase concentration or unreacted precursors. The XRD pattern of OAM 0.1 sample is presented in Figure S4 (Supporting Information). The XRD pattern confirms the similar cubic phase of CsPbBr_3_ as OAM 0.4.

### 3.2 Absorbance and Photoluminescence Studies

**Figure 3:**
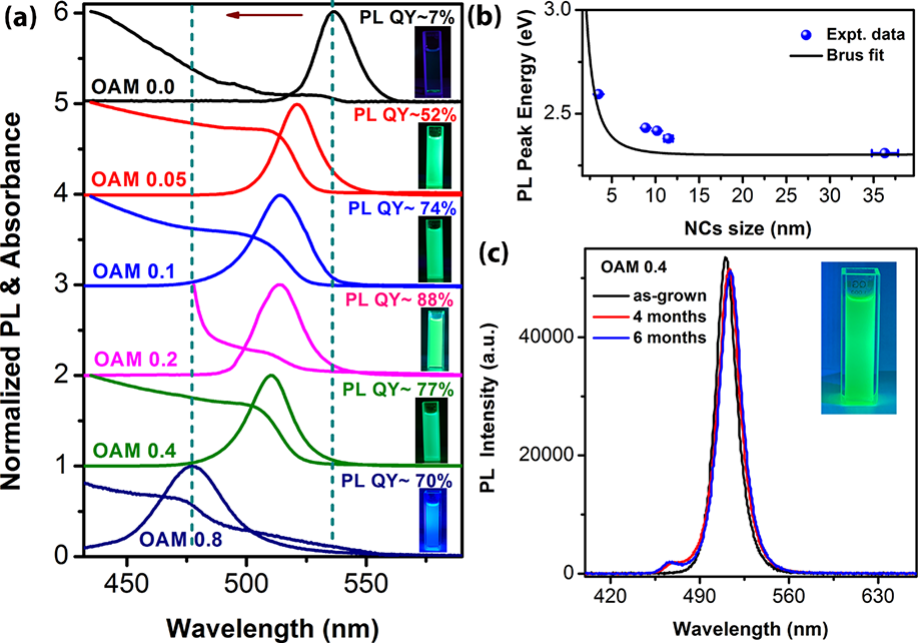
(a) Comparison of absorption edge and PL emission of CsPbBr_3_ NCs synthesized with different amounts of oleylamine. The right inset shows the emissions of the colloidal NCs under UV light. (b) Variation PL peak energy with the NCs size of different samples obtained from TEM images. The solid line corresponds Brus fit of CsPbBr_3_ using the quantum confinement model. (c) Comparison of PL intensity of as-grown colloidal dispersion of OAM 0.4 NCs and after 4, 6 months of ambient storage. Inset shows the scintillation of the NCs under UV illumination, after 6 months of storage under ambient conditions, confirming their excellent stability.

Figure 3a shows the comparison of absorption and PL spectra of CsPbBr_3_ NCs synthesized with 0, 0.05, 0.1, 0.2, 0.4, and 0.8 mL of OAM. The absorption edge and the PL emission peaks were blue-shifted with increasing OAM concentration, which is consistent with quantum confinement effects due to decreasing particle size, as shown in the TEM images. Figure S5 (Supporting Information) presents the normalized PL peak of different samples showing the blue-shift of peak emission wavelength. For a quantitative assessment of the quantum confinement effect and blue-shift of the PL peak, the PL peak energy was plotted versus NCs size along with the well-known Brus fit (solid line) as shown in Figure 3b using the formula:^39,40^
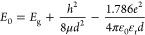
1where *E*_0_ is the energy of the lowest excited state of the exciton inside the NCs/QDs, *E*_g_ is the bandgap of bulk CsPbBr_3_ (∼2.3 eV), *h* is Planck’s constant, μ is the reduced mass of CsPbBr_3_ (0.12*m*_0_), *e* is the electron charge, ε_r_ is the dielectric constant of CsPbBr_3_ (ε_r_ = 7.3), and *d* is the size of the NCs.^41,42^ Interestingly, the experimentally calculated PL peak energies of different NCs are very close to the Brus fit, confirming the quantum confinement of excitons (Figure 3b).

The PLQY was observed to be 7, 52, 74, 88, 77, and 70% in NCs synthesized with 0, 0.05, 0.1, 0.2, 0.4, and 0.8 mL of OAM, respectively. The PLQY of the CsPbBr_3_ NCs grown by the facile mechanochemical method is comparable to the well-known hot injection method.^43^ In terms of optimizing the light emission, the PLQY was significantly increased with the increase in OAM surfactant due to improved surface functionalization and formation of NCs. The inset of Figure S5 (Supporting Information) shows the variation of PLQY with the OAM content used during synthesis. The OAM ligand on the surface of the perovskite NCs plays an important role in both the luminescence properties and the NC stability. However, with the use of excess OAM (beyond 0.2 mL), the PLQY was further decreased, which may be attributed to higher surface capping by the ligand. The excessive surface ligands act as an insulation layer which may cause light scattering and lesser PL yield.^44^ There is also a possibility of the formation of an amide from the excess OAM which may destabilize the colloidal NCs.^45^ The OAM also plays an important role in reducing the aggregation and self-assembly of CsPbBr_3_ NCs when cast into PMMA to form a composite scintillator.^25^ The right inset of Figure 3a shows the photographs of colloidal CsPbBr_3_ NCs with strong green emission under UV light.

We further tested the stability of our NCs by PL measurement. Figure 3c shows the comparison of PL intensities of as-grown OAM 0.4 NCs and after 4 and 6 months of ambient storage. The inset of Figure 3c depicts the photograph of 6 months of stored NCs under UV light showing strong green emission. The comparison of PL spectra of as-grown OAM 0.2 NCs after 4 months of ambient storage is presented in Figure S6 (Supporting Information). The NCs grown by the mechanosynthesis method exhibit excellent stability, which may be aided by the polar solvent-free, solid-state synthesis process.^46,47^

**Figure 4:**
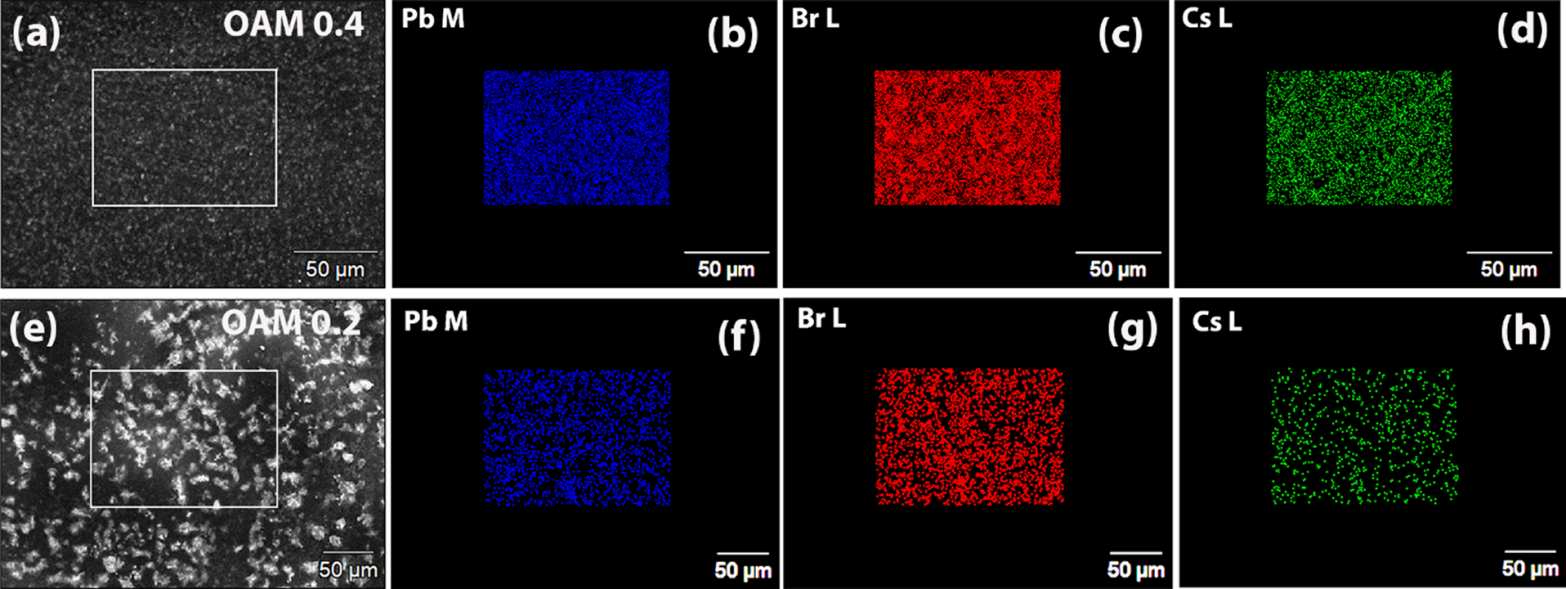
(a) SEM image of CsPbBr_3_ NCs with 0.4 mL of OAM/PMMA disc. (b–d) Corresponding EDS elemental mapping of the selected portion of Figure 4a. (e) SEM image of CsPbBr_3_ NCs with 0.2 mL of OAM/PMMA disc. (f–h) Corresponding EDS elemental mapping of the selected portion of Figure 4e.

The colloidal dispersions of CsPbBr_3_ NCs synthesized with different amounts of OAM were mixed with PMMA polymer and cast into discs of 2 mm thickness and 4 cm diameter using PTFE molds. The SEM images of the surface of CsPbBr_3_/PMMA composites with OAM 0.4 and OAM 0.2 are shown in Figure 4a and Figure 4e, respectively, while the corresponding EDS elemental mapping is shown in Figure 4b–d and Figure 4f–h. Interestingly, larger sizes of agglomerated perovskite particles were observed in the composite disc with OAM 0.2, whereas in the case of OAM 0.4, the perovskite spatial distribution was very uniform. During mixing, casting, and solidification, CsPbBr_3_ NCs tend to self-assemble and agglomerate into bigger particles which can further quench the optical emission properties.^48^ The higher concentration of OAM capped on the surface of the NCs in sample OAM 0.4 prevents agglomeration, which results in excellent PL and RL emission.^49^ However, a further increase in OAM (0.8 mL) during mechanochemical synthesis leads to NCs with lower PLQY.

**Figure 5:**
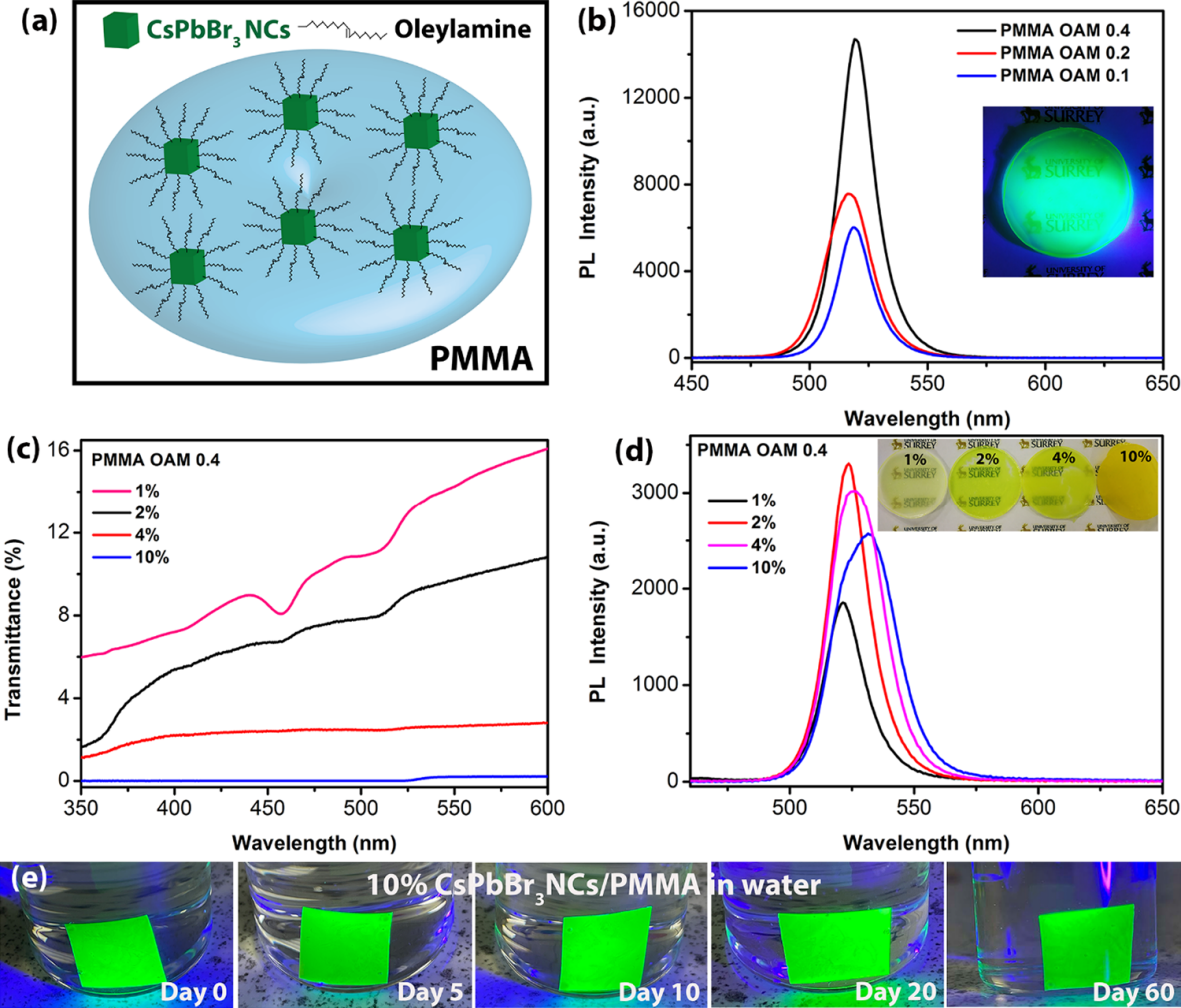
(a) Schematic illustration of oleylamine capped CsPbBr_3_ NCs embedded in PMMM disc. Oleylamine on top of the NCs prevents aggregation and hence the decrease in emission properties. (b) Comparison of PL of 2% CsPbBr_3_ NCs loaded PMMA disc with different amounts of OAM. Inset shows the emission of a disc under UV light. (c) Variation of transmittance of 1, 2, 4, and 10% CsPbBr_3_ loaded PMMA disc with 0.4 mL of OAM. (d) Comparison of PL of 1, 2, 4, and 10% CsPbBr_3_ loaded PMMA disc with 0.4 mL of OAM. Inset shows a photograph of different CsPbBr_3_-loaded PMMA discs. (e) Stability test of CsPbBr_3_/PMMA nanocomposite in water. The nanocomposite exhibits intense emission after 60 days of storage in water.

A schematic illustration of OAM-capped CsPbBr_3_ NCs embedded in PMMA is depicted in Figure 5a. Figure 5b shows the comparison of PL spectra of CsPbBr_3_/PMMA composites with OAM 0.1, 0.2, and 0.4. All the samples show strong emission with a peak emission wavelength of ∼519 nm due to the band edge excitonic recombination.^50^ There is a small blue shift of PL peak position in the nanocomposite disc with OMA 0.2 compared to those of OMA 0.1 and OMA 0.4. This shift may be due to local variation in the nanocomposite composition or thickness that affects the optical reabsorption phenomenon. The PMMA OAM 0.4 disc exhibits the highest PL emission, which is attributed to less agglomeration and PL quenching. A similar trend in the RL emission properties was also observed. However, OAM 0.2 shows the highest PLQY in colloidal form. The inset of Figure 5b depicts an image of the CsPbBr_3_/PMMA nanocomposite disc under UV light showing bright green emission.

In order to optimize the PL and scintillation properties of the composite, we varied the perovskite mass loading in the nanocomposite disc from 1 to 10% with OAM 0.4 NCs. Higher loading of NCs containing high-Z atoms will produce a greater interaction with the incident X-rays and hence a larger detection efficiency.^51,52^ However, the optical emission yield may be compromised due to greater optical scattering in those composites with higher percentage loading. The optical transmission of the CsPbBr_3_/PMMA composite decreases with an increase in loading concentration due to higher absorbance and scattering, as shown in Figure 5c. A sharp decrease in transmission near 530 nm was observed for all the samples, which corresponds to the band edge absorbance of CsPbBr_3_. Figure 5d shows the comparison of PL spectra of PMMA composites with 1, 2, 4, and 10% loading of CsPbBr_3_. The inset of Figure 5d depicts an image of perovskite-loaded PMMA discs with increasing NCs loading. Interestingly the 2% CsPbBr_3_/PMMA disc exhibits the highest PL emission due to optimum scattering and self-absorption. However, the 10% CsPbBr_3_ loaded disc exhibits the strongest RL emission due to the greater X-ray attenuation from the higher concentration of NCs. With the increase in perovskite loading concentration, the PL peak was slightly red-shifted. This may be due to the agglomeration and formation of larger-size NCs at the higher perovskite loading during mixing and casting into CsPbBr_3_/PMMA nanocomposite scintillator discs.

One of the major shortcomings of perovskite materials and devices is poor stability in the presence of oxygen and water.^53−55^ Interestingly, the CsPbBr_3_/PMMA nanocomposite exhibits excellent water resistance and stability, which is attributed to the encapsulation of the NCs by the stable PMMA matrix.^40,56,57^Figure 5e shows photographs of 10% CsPbBr_3_ NC-loaded PMMA nanocomposite dipped in water under UV light. No visible fluorescence quenching was observed for up to 60 days of storage in water which confirms the robustness of the nanocomposite. The high stability may also be due to the unique solvent-free synthesis strategy of the NCs.

**Figure 6:**
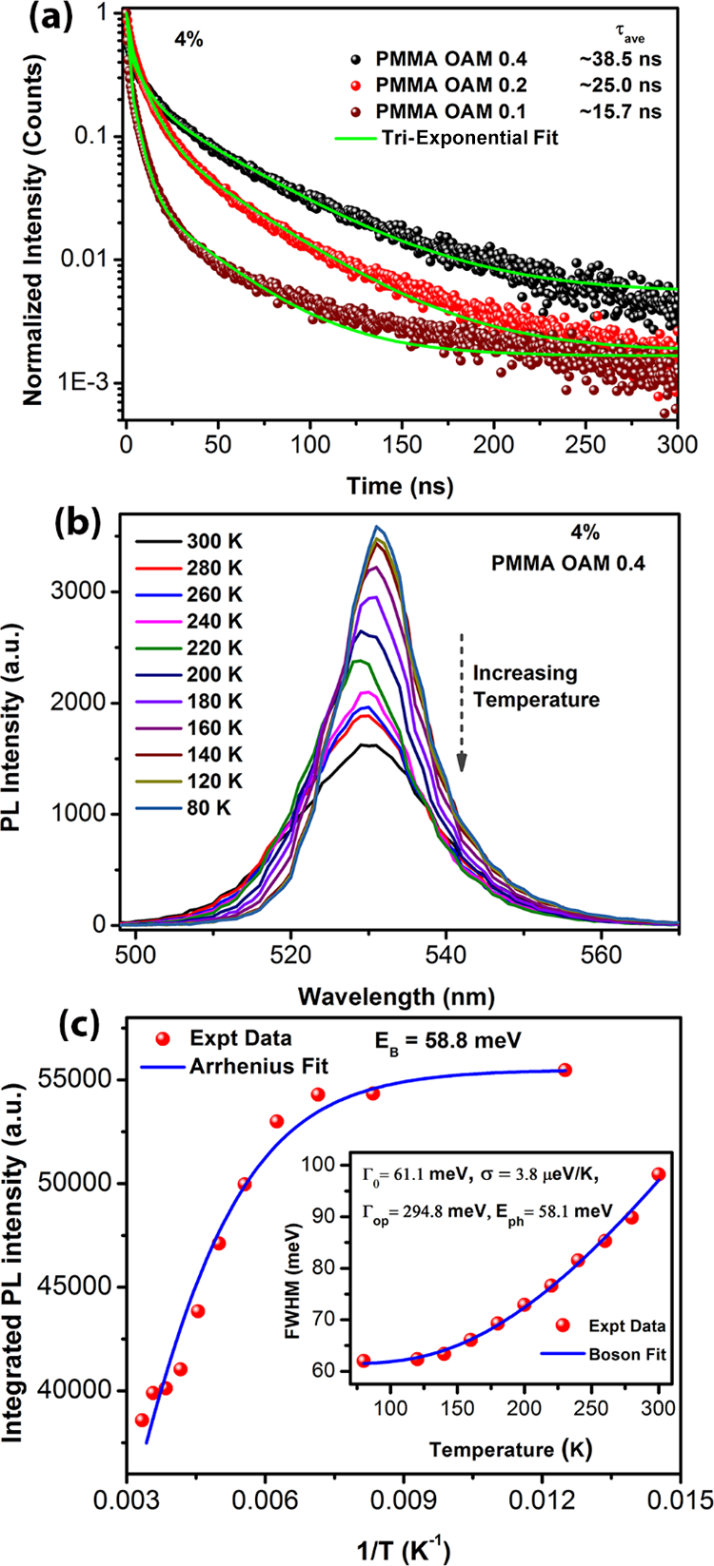
(a) TRPL decay profiles with the tri-exponential fit of CsPbBr3 NCs synthesized with different amounts of OAM. (b) Temperature-dependent PL spectra of 10% CsPbBr_3_ loaded PMMA disc with 0.4 mL of OAM. (c) Integrated PL intensity as a function of the inverse of temperature and its fitting with the Arrhenius-like equation. Inset shows the variation of FWHM of the PL peak with temperatures with Boson fit.

To investigate the photo carrier recombination kinetics of the CsPbBr_3_ NCs embedded in the PMMA matrix, we performed time-resolved photoluminescence (TRPL) measurements. Figure 6a shows the TRPL decay profiles of different CsPbBr_3_/PMMA composites fitted using a tri-exponential decay function.^57,58^ The time constants and relative weights of the TRPL decay profiles are tabulated in Table S1 (Supporting Information). The tri-exponential decay times are attributed to band-edge excitonic recombination, shallow trap-mediated radiative recombination, and the trap states arising from the surface defects.^59,60^ The average lifetimes (τ_ave_) were calculated to be 15.7, 25.0, and 38.5 ns for composites with OAM 0.1, 0.2, and 0.4 samples, respectively. The increase in recombination decay times with the increase in OAM may be attributed to the removal of nonradiative decay pathways with the increase in OAM content.^61^ The TRPL decay profiles of the nanocomposite scintillators with different CsPbBr_3_ NCs loading concentrations are shown in Figure S7 (Supporting Information). The average decay times obtained were between 38 and 55 ns. The decay time was shortened in the 10% perovskite-loaded nanocomposite which may be due to higher radiative recombination.

We performed a temperature-dependent PL study of the perovskite/PMMA composite to estimate the exciton binding energy (*E*_B_) of the embedded CsPbBr_3_ NCs. The PL peak intensity decreased with the increase in temperature from 80 to 300 K due to thermal quenching and carrier trapping at higher temperatures (Figure 6b). The nonradiative deep-level trap states are more effective, and excitons dissociate at higher temperatures, which leads to a decrease in PL intensity.^62^ To estimate the exciton binding energy (*E*_B_) of CsPbBr_3_ NCs in PMMA, we have fitted the integrated PL peak intensity versus the inverse of temperature (1/*T*) (Figure 6c) using an Arrhenius equation as given by^62^
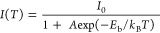
2where *I*_0_ is the integrated PL intensity at low temperatures, *E*_b_ is the exciton binding energy, and *k*_B_ is the Boltzmann constant. From our data, the excitation binding energy was estimated to be 58.8 meV, which is consistent with the previous reports for colloidal CsPbBr_3_ NCs and supports the high PL emission intensity observed at room temperature.^63^

The FWHM of the PL peak increases with the increase in temperature, which is attributed to the enhanced exciton-phonon scattering at higher temperatures. We have fitted the linewidth broadening using the Boson model with the following equation:
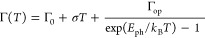
3where *Γ*_0_represents an inhomogeneous broadening constant, Γ_op_ is the exciton-longitudinal optical phonon coupling coefficient, σ presents the exciton-acoustic phonon coupling coefficient, and *E*_ph_ is the optical phonon energy. From the fitting of the data in the inset of Figure 6c, the obtained parameters were Γ_0_ = 61.1 meV, Γ_op_ = 294.8 meV, σ = 3.8 μeV/K, and *E*_ph_ = 58.8 meV. The calculated values of CsPbBr_3_ NCs embedded in PMMA are similar to the earlier report on CsPbBr_3_ NC film.^50^

### 3.3 Scintillation Performance

**Figure 7:**
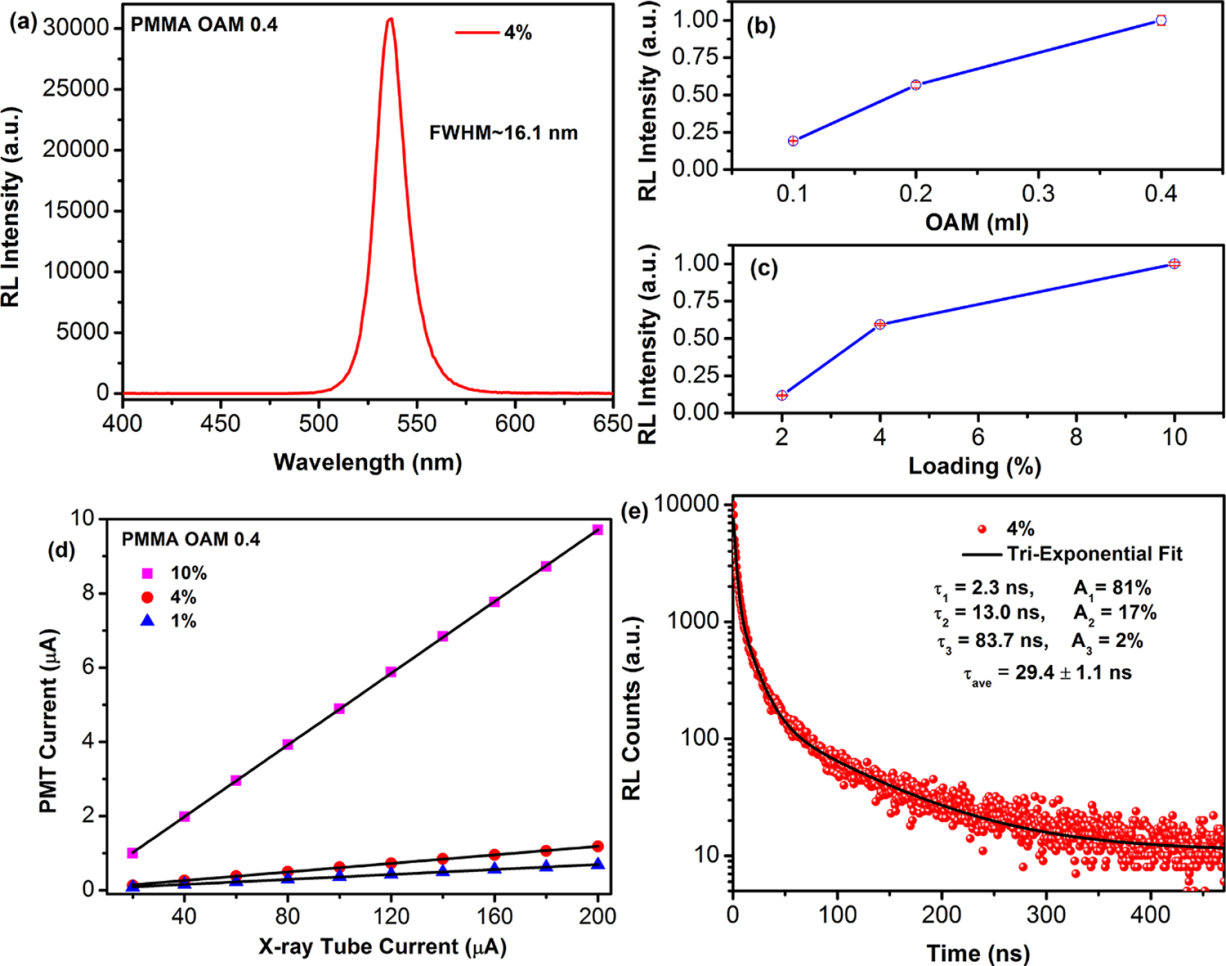
(a) RL emission spectrum of CsPbBr_3_/PMMA nanocomposite. (b) Variation of RL intensity of nanocomposite with OAM used during the synthesis of the NCs. (c) Variation of RL intensity with NCs loading percentage. (d) PMMT current obtained from different perovskite loaded discs at varying dose rates. (e) RL decay profile of 4% CsPbBr_3_ NCs loaded nanocomposite disc with the tri-exponential fit.

Considering the high X-ray attenuation of CsPbBr_3_ and strong emission properties with excellent stability, the perovskite PMMA nanocomposite is a potential candidate for low-cost X-ray detection and imaging. Figure 7a shows the RL emission spectra of the 4% CsPbBr_3_ NC loaded PMMA disc under excitation by a 60 kV X-ray with a tube current of 100 μA. Strong green emission with a peak at 536 nm and an FWHM of 16.1 nm was observed.^1,19^ The RL emission peak matches well with the excitonic PL emission peak and absorption edge. A small red shift in the RL peak was observed as compared to the PL peak which may be attributed to the self-absorption effects. Note that the green RL emission band is in the response range of commercial PMTs and CCD cameras, which makes these nanocomposites useful as potential scintillators-based detectors. The RL peak intensity of the CsPbBr_3_/PMMA nanocomposite increased with an increase in OAM content during synthesis (Figure 7b), which is similar to the PL analysis. With the increase in OAM, less agglomeration of CsPbBr_3_ NCs embedded in PMMA was observed, which resulted in higher PL/RL emission intensity. The RL peak intensity also increases with the increase in CsPbBr_3_ NCs loading from 2 to 10% in PMMA (Figure 7c). However, the 2% CsPbBr_3_/PMMA nanocomposite shows the PL emission with a higher intensity as compared to the 10% CsPbBr_3_ loaded disc. The PL emission comes from near the surface of the composite disc, and light scattering increases with higher loading which may result in a decrease in PL for 10% CsPbBr_3_/PMMA disc. However, the significantly deeper penetration of X-rays into the composite material excites a higher volume of perovskite which produces a greater RL intensity in composites with greater percentage loading. The X-ray detection efficiency of composites with higher NC loading tends to dominate the RL performance, with a trade-off at high loading between the greater detection efficiency and the reduced optical transmission.

The light emission response of the different perovskite-loaded PMMA discs was tested using a photomultiplier tube (PMT) under different X-ray dose rates with a tube voltage of 40 kV and the X-ray tube current varied from 20 to 200 μA (Figure 7d). The observed linear response of the PMT photocurrent as a function of X-ray tube current confirms the suitability of CsPbBr_3_/PMMA nanocomposites for sensitive and quantitative X-ray imaging. The 10% loaded CsPbBr_3_/PMMA disc showed a higher PMT current due to the higher interaction of perovskite NCs with X-rays, which is consistent with the RL study. The light yield of the 10% CsPbBr_3_ NCs loaded PMMA disc was compared to a 2 mm-thick commercial LYSO:Ce scintillator under different X-ray dose rates with a tube voltage of 40 kV and the X-ray tube current varied from 20 to 200 μA. Figure S8 (Supporting Information) shows that the PMT photocurrent from the 10% CsPbBr_3_ NC loaded PMMA disc is 41% of the photocurrent from the LYSO:Ce scintillator of similar thickness. This confirms the high brightness of the CsPbBr_3_/PMMA nanocomposite, given that it is only 10% loaded with perovskite NCs.

To estimate the scintillation time response of the nanocomposite, RL decay time measurements were performed using a pulsed X-ray source (Figure 7e). The RL decay profile of the 4% CsPbBr_3_ NC-loaded PMMA disc was fitted with a tri-exponential decay function, and time constants with relative weights are shown in the inset of Figure 7e.^16,64^ The shorter time constant with maximum weight corresponds to the X-ray excited excitonic recombination, while the longer decay constants may be associated with trap states arising from the surface defects and shallow trap-mediated radiative recombination.^59,60^ The average RL decay time obtained was 29.4 ns which is significantly faster than current commercial X-ray imaging scintillators such as CsI:Tl (∼680 ns decay time).

**Figure 8:**
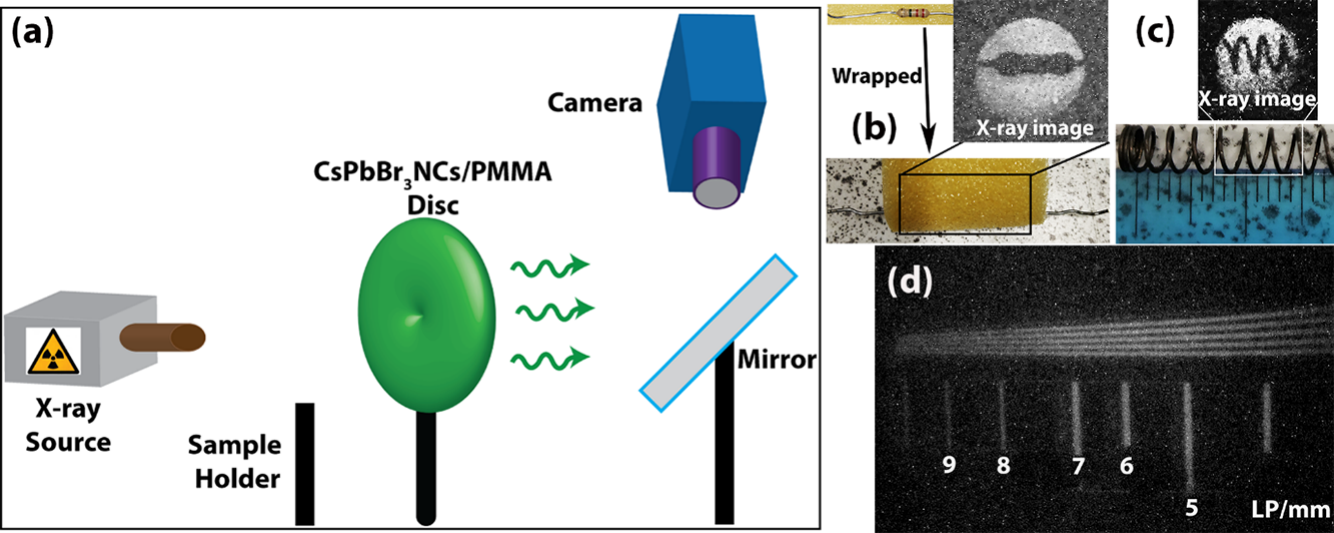
(a) Schematic illustration of X-ray imaging setup using CsPbBr_3_/PMMA nanocomposite disc as a scintillator. (b) Bright-field and an X-ray image of a wrapped resistor. (c) Bright-field and an X-ray image of a spring of 0.4 mm diameter. (d) X-ray image of a standard X-ray resolution test pattern plate.

As a proof-of-concept experiment, a 10% CsPbBr_3_ NC-loaded PMMA scintillator disc of diameter of 4 cm and thickness of 2 mm was used for X-ray imaging.^65,66^Figure 8a depicts the schematic illustration of the X-ray imaging setup, which comprises an X-ray source, a test sample, a CsPbBr_3_/PMMA nanocomposite scintillator disc, a mirror, and a commercial CCD camera. The inset of Figure 8b shows an X-ray image obtained using the scintillator disc of a resistor wrapped with an opaque sheet (Figure 8b). The inset of Figure 8c depicts the X-ray image of a 0.4 mm spring from a pen, which confirms the good resolution of the nanocomposite disc. To measure the spatial resolution ability of our prototype, we further performed imaging of a standard X-ray test-pattern plate, as shown in Figure 8d.^67^ The observable X-ray imaging resolution of the 10% CsPbBr_3_ NC loaded PMMA scintillator disc was ∼8 lp/mm. Thus, the nanocomposite scintillator can produce a good-quality X-ray image, holding the potential for low-cost sensitive radiography. In Table S2 (Supporting Information), we have compared the resolution of the CsPbBr_3_/PMMA nanocomposite scintillator with other reported perovskite scintillator and commercial CsI and LYSO:Ce scintillators. Our nanocomposite scintillator exhibits comparable resolution to the reported perovskite scintillators. However, further research is required to achieve the resolution of the commercial scintillator.

## 4 Conclusions

We have demonstrated a facile surfactant-dependent solid-state synthesis of highly luminescent CsPbBr_3_ NCs and studied their scintillation properties. The solid-state synthesized perovskite NCs exhibit a high photoluminescence quantum yield of up to 88% with excellent stability. CsPbBr_3_ perovskite NCs dispersions capped with different amounts of surfactant were mixed with PMMA plastic and cast into discs of 2 mm thickness. The higher PL yield of the CsPbBr_3_/PMMA nanocomposite with an increase in OAM during synthesis is attributed to decreased aggregation and reduced PL quenching. OAM on the surface of the NCs helps in reducing self-assembly and aggregation in the composite. We also varied the perovskite loading concentration in the nanocomposites and studied the resulting emission properties. The strongest radioluminescence emission was observed in a 10% CsPbBr_3_/PMMA nanocomposite disc, while the highest PL emission was obtained in a 2% perovskite-loaded PMMA disc. This may be attributed to the high penetration and interaction of the X-rays through the entire thickness of the nanocomposite disc, whereas PL comes optical absorption from the near surface of the disc. The CsPbBr_3_/PMMA nanocomposite disc exhibits a highly intense RL emission peak at 536 nm with FWHM ∼16 nm with a fast RL decay time of 29.4 ns. Further, we have demonstrated X-ray imaging using the 10% CsPbBr_3_ NCs loaded PMMA nanocomposite disc with high spatial resolution. Our results open up the possibility of CsPbBr_3_/PMMA nanocomposite for low-cost ionizing detection and imaging.

## Supporting Information Available

Tables containing TRPL decay constants with weightages (Table S1); comparison table of resolution of the scintillator (Table S2); TEM images of samples OAM 0, OAM 0.05, OAM 0.1 with corresponding particle size distributions (Figure S1); SAED pattern of CsPbBr_3_ NC (Figure S2); XRD pattern of OAM 0.4 sample (Figure S3); XRD pattern of OAM 0.4 sample (Figure S4); normalized PL spectra of CsPbBr_3_ NCs synthesized with different amount of OAM (Figure S5); PL spectra of as-grown OAM 0.2 NCs and after 4 months of ambient storage (Figure S6); TRPL decay profiles of different CsPbBr_3_ NCs/PMMA nanocomposite (Figure S7); comparison of PMT current obtained from 10% CsPbBr_3_ NCs/PMMA and LYSO:Ce scintillators (Figure S8) (PDF)
