# Supplementary material for: Surfactant-Dependent Bulk Scale Mechanochemical Synthesis of CsPbBr3 Nanocrystals for Plastic Scintillator-Based X-ray Imaging
Source: ACS Appl Nano Mater. 2023 Aug 7;6(16):14980–90. doi: 10.1021/acsanm.3c02531 (PMC10463220; doi:10.1021/acsanm.3c02531)
Supplement: Supplementary file 1 — an3c02531_si_001.pdf [file an3c02531_si_001.pdf]

Supporting Information

Surfactant Dependent Bulk Scale Mechanochemical Synthesis of $CsPbBr_3$ Nanocrystals for Plastic Scintillator-Based X-ray Imaging

Joydip Ghosh[1*], Joseph O'Neill[1], Mateus G. Masteghin[2], Isabel Braddock[1], Carol Crean[3], Robert Dorey[4], Hayden Salway[5], Miguel Anaya[5], Justin Reiss[6], Douglas Wolfe[6], and Paul Sellin[1*]

[1]*Department of Physics, University of Surrey, Guildford GU2 7XH, Surrey, UK*

[2]*Advanced Technology Institute, University of Surrey, Guildford GU2 7XH, UK*

[3]*Department of Chemistry, University of Surrey, Guildford GU2 7XH, UK*

[4]*School of Mechanical Engineering Sciences, University of Surrey, Guildford GU2 7XH, UK*

[5]*Department of Chemical Engineering and Biotechnology, University of Cambridge, CB3 0AS, UK*

[6]*Applied Research Laboratory, Materials Science and Engineering Department, The Pennsylvania State University, University Park, PA 16802, USA*

[*] Corresponding authors, email, J.G.: j.ghosh@surrey.ac.uk, P.S.: p.sellin@surrey.ac.uk

Table S1: Details of the fitting parameters of TRPL decay for $CsPbBr_3$ NCs synthesized with different OAM concentrations.

Sample	τ_1 (ns)	A_1 (%)	τ_2 (ns)	A_2 (%)	τ_3 (ns)	A_3 (%)	τ_{ave} (ns)
OAM 0.1	0.5	60.2	4.8	35.9	34.4	3.8	15.7
OAM 0.2	1.5	33.7	8.3	53.7	42.4	12.2	25.0
OAM 0.4	0.74	48	8.2	32	47.8	20	38.5

Table S2: Comparison of the X-ray resolution of the scintillators.

scintillators	Resolution	Reference
Li-doped PEA_2PbBr_4 (PEA= phenethylammonium)	8.8 at 0.2 MTF	[1]
CsPbBrNCs	2 lp/mm at 0.72 MTF	[2]
TPP_2MnBr_4 (TPP =tetraphe-nylphosphonium)	15.7 lp/mm at 0.2 MTF	[3]
$Cs_3Cu_2I_5$ film	17 lp/mm at 0.2 MTF	[4]
CsI:Tl	100 lp/mm at 0.1 MTF)	[5]
LYSO:Ce	2.5 lp/mm	[6]
CsPbBr3/PMMA nanocomposite	~8 lp/mm	This study

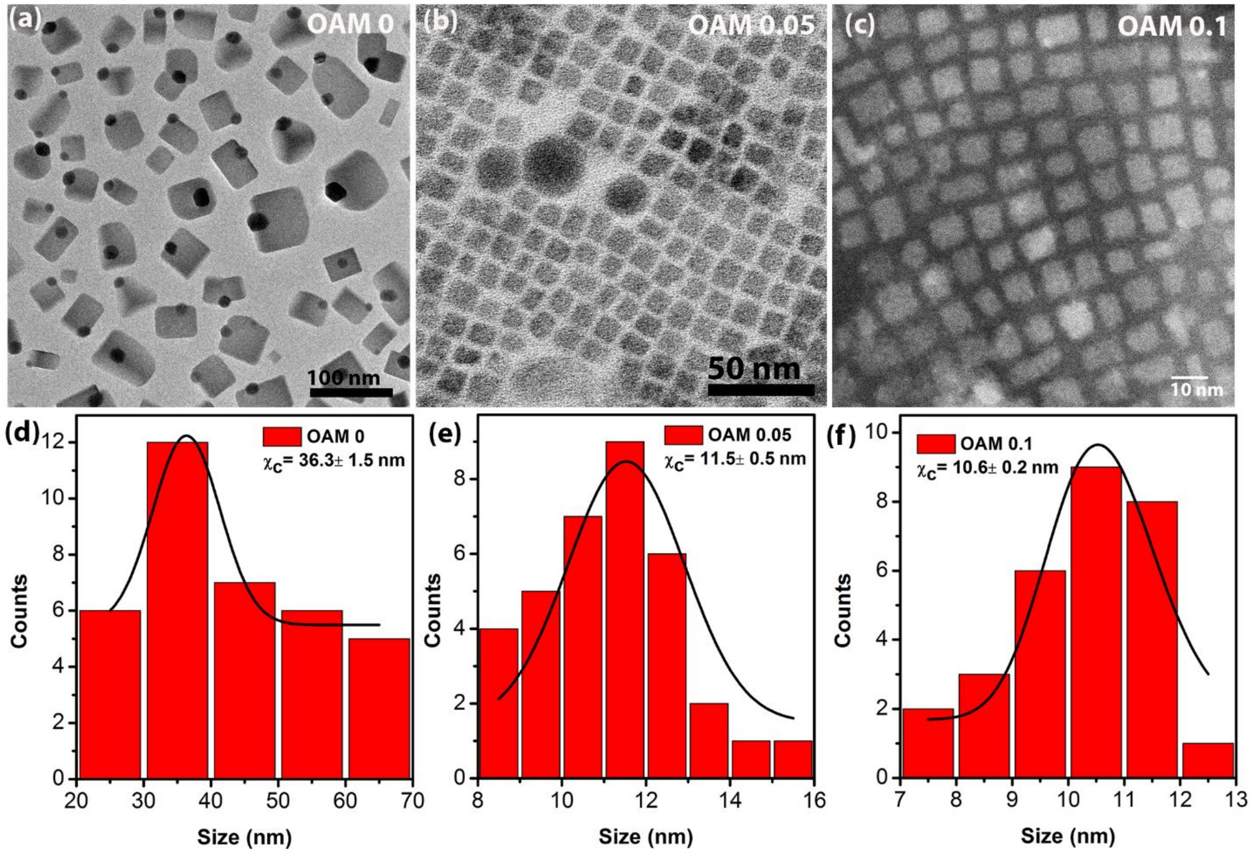

Fig. S1: TEM images of $CsPbBr_3$ NCs synthesized with (a) 0 ml of OAM, (b) 0.05 ml of OAM, (c) 0.1 ml of OAM, (d) particle size distribution of OAM 0 sample, (e) particle size distribution of OAM 0.05 sample, (f) particle size distribution of OAM 0.1 sample.

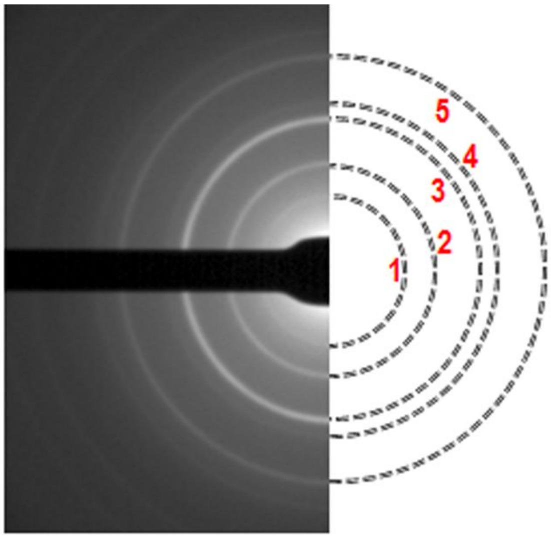

	1	2	3	4	5
d (nm)	5.82	4.13	2.94	2.62	2.07
(hkl)	(100)	(110)	(002)	(210)	(202)

Fig. S2: SAED pattern of $CsPbBr_3$ NC indexed based on the PDF #18-364.

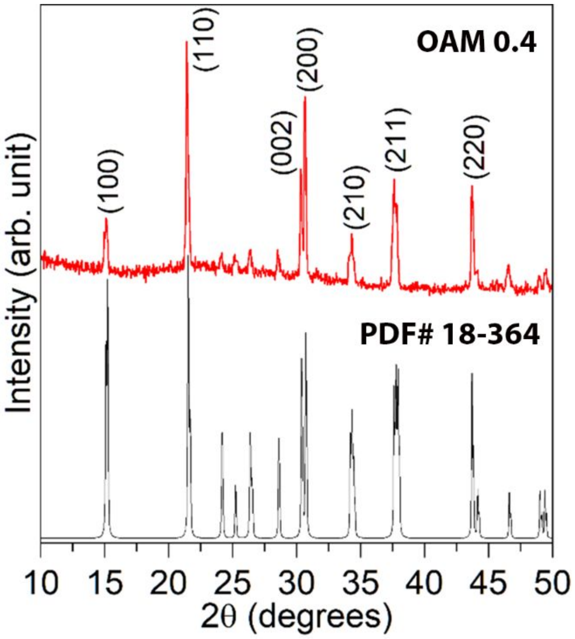

Fig. S3: XRD pattern of OAM 0.4 sample. Red-colored line corresponds to the experimental diffraction pattern while the black-colored line is the standard pattern from PDF #18-364 .

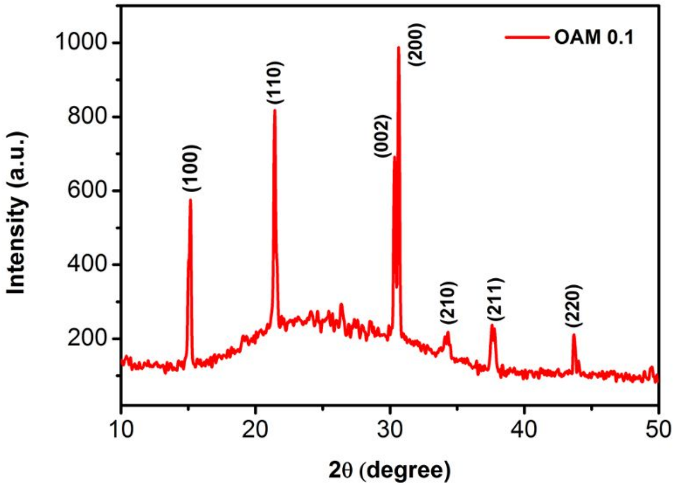

Fig. S4: XRD pattern of OAM 0.1 sample.

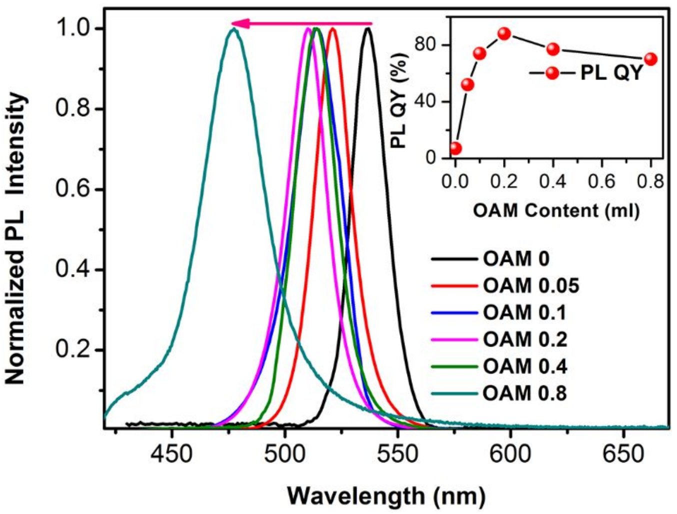

Fig. S5: Normalized PL emission spectra of $CsPbBr_3$ NCs synthesized with different amount of OAM. The inset shows the variation of PL QY with OAM content used during synthesis.

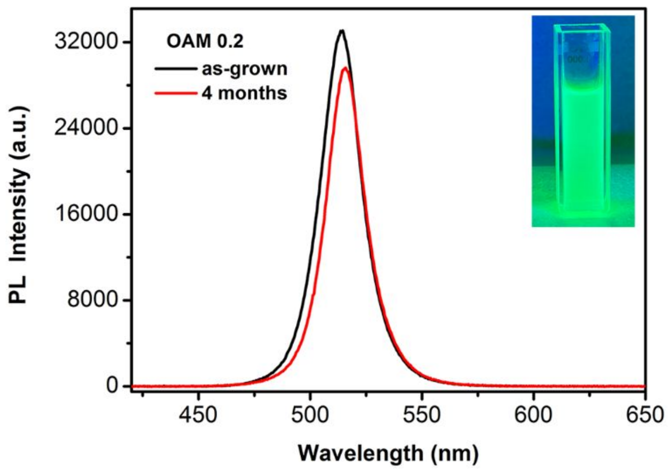

Fig. S6: Comparison of PL spectra of as-grown colloidal dispersion of OAM 0.2 NCs and after four months of ambient storage. Inset shows the scintillation of the NCs under UV illumination, after four months of storage in ambient conditions, confirming their excellent stability.

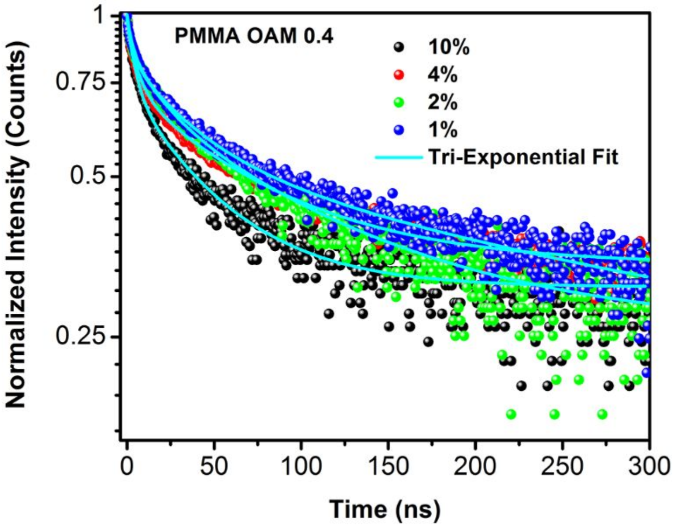

Fig. S7: TRPL decay profiles of the nanocomposite scintillators with different $CsPbBr_3$ NCs loading.

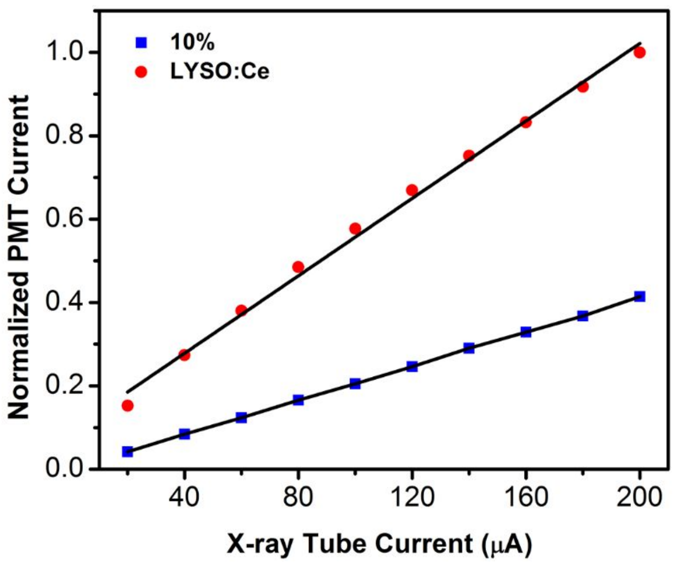

Fig. S8: Comparison of PMMT current obtained from 10% $CsPbBr_3$ NCs/PMMA and LYSO:Ce scintillators at varying X-ray tube currents.

References:

(1) Datta, A.; Fiala, J.; Motakef, S. 2D perovskite-based high spatial resolution X-ray detectors. *Sci. Rep.* **2021**, *11*, 22897.

(2) Chen, Q.; Wu, J.; Ou, X.; Huang, B.; Almutlaq, J.; Zhumekenov, A. A.; Guan, X.; Han, S.; Liang, L.; Yi, Z.; Li, J.; Xie, X.; Wang, Y.; Li, Y.; Fan, D.; Teh, D. B. L.; All, A. H.; Mohammed, O. F.; Bakr, O. M.; Wu, T.; Bettinelli, M.; Yang, H.; Huang, W.; Liu, X. All-inorganic perovskite nanocrystal scintillators. *Nature* **2018**, *561*, 88-93.

(3) Han, K.; Sakhatskyi, K.; Jin, J.; Zhang, Q.; Kovalenko, M. V.; Xia, Z. Seed-Crystal-Induced Cold Sintering Toward Metal Halide Transparent Ceramic Scintillators. *Adv. Mater.* **2022**, *34*, 2110420.

(4) Zhou, Y.; Wang, X.; He, T.; Yang, H.; Yang, C.; Shao, B.; Gutiérrez-Arzaluz, L.; Bakr, O. M.; Zhang, Y.; Mohammed, O. F. Large-Area Perovskite-Related Copper Halide Film for High-Resolution Flexible X-ray Imaging Scintillation Screens. *ACS Energy Lett.* **2022**, *7*, 844-846.

(5) Hormozan, Y.; Sychugov, I.; Linnros, J. High-resolution x-ray imaging using a structured scintillator. *Med. Phys.* **2016**, *43*, 696-701.

(6) Yan, W.; Li, B.; Duan, B.; Song, G.; Song, Y.; Ma, J. Temperature dependence of luminescence characteristics of LYSO:Ce scintillator under x-ray excitation. *AIP Advances* **2022**, *12*.
